# Care Models of eHealth Services: A Case Study on the Design of a Business Model for an Online Precare Service

**DOI:** 10.2196/resprot.3501

**Published:** 2015-03-24

**Authors:** Dorine PD van Meeuwen, Quirine J van Walt Meijer, Lianne WL Simonse

**Affiliations:** ^1^Product Innovation Management departmentFaculty of Industrial Design EngineeringDelft University of TechnologyDelftNetherlands

**Keywords:** eHealth, business model innovation, strategic design, precare, service design, visual modeling method, care model

## Abstract

**Background:**

With a growing population of health care clients in the future, the organization of high-quality and cost-effective service providing becomes an increasing challenge. New online eHealth services are proposed as innovative options for the future. Yet, a major barrier to these services appears to be the lack of new business model designs. Although design efforts generally result in visual models, no such artifacts have been found in the literature on business model design. This paper investigates business model design in eHealth service practices from a design perspective. It adopts a research by design approach and seeks to unravel what characteristics of business models determine an online service and what are important value exchanges between health professionals and clients.

**Objective:**

The objective of the study was to analyze the construction of care models in-depth, framing the essential elements of a business model, and design a new care model that structures these elements for the particular context of an online pre-care service in practice.

**Methods:**

This research employs a qualitative method of an in-depth case study in which different perspectives on constructing a care model are investigated. Data are collected by using the visual business modeling toolkit, designed to cocreate and visualize the business model. The cocreated models are transcribed and analyzed per actor perspective, transactions, and value attributes.

**Results:**

We revealed eight new actors in the business model for providing the service. Essential actors are: the intermediary network coordinator connecting companies, the service dedicated information technology specialists, and the service dedicated health specialist. In the transactions for every service providing we found a certain type of contract, such as a license contract and service contracts for precare services and software products. In addition to the efficiency, quality, and convenience, important value attributes appeared to be: timelines, privacy and credibility, availability, pleasantness, and social interaction. Based on the in-depth insights from the actor perspectives, the business model for online precare services is modeled with a visual design. A new care model of the online precare service is designed and compiled of building blocks for the business model.

**Conclusions:**

For the construction of a care model, actors, transactions, and value attributes are essential elements. The design of a care model structures these elements in a visual way. Guided by the business modeling toolkit, the care model design artifact is visualized in the context of an online precare service. Important building blocks include: provision of an online flow of information with regular interactions to the client stimulates self-management of personal health and service-dedicated health expert ensure an increase of the perceived quality of the eHealth service.

## Introduction

### Implementation Barrier of eHealth Services

Innovative eHealth technologies have the potential to provide solutions for several challenges within health care, including the growing number of chronic diseases, rising shortage of health staff, and the pressure to increase cost savings within healthcare [1-4]. An illustrative example is an eHealth service for diabetes that provides state-of-the-art technologies that have the potential to assist health care professionals, patients, and informal carers to better manage diabetes insulin therapy, help patients understand their disease, support self-management, and provide a safe environment. Yet, in order to realize these potential benefits, new designs of care models are required [5-7].

A growing number of scholars at the intersection of medical informatics, public health, and business are investigating eHealth and related technologies [3]. The Internet and mobile applications inspire new business model innovations. These Internet technologies lead to drastic changes in the way that organizations interact [8]. Despite the growing awareness of the importance of business model design, the nature of business model design has received little attention. To date, research has concentrated on piloting Internet and related technologies in diverse care and cure contexts, with less attention paid to the business interactions between organizations. There is a lack of in-depth knowledge on the network organizations influencing eHealth services. Most studies have targeted the care and cure phase of the health care chain. So far, academic research has not emphasized business model design in this early phase of the health care chain. To contribute to this research area, the present paper aims to generate an improved understanding of how to design a business model in order to overcome the implementation barrier of eHealth services; in particular, we seek to arrive at an in-depth understanding of the value exchanges within precare service interactions. This paper adopts the following definition of precare, “health-protective behavior before the actual care phase” [9].

### Theoretical Background on Business Model Design

Business model design was conceived when online services such as those provided by Amazon were established, and new constructs were needed for the purpose of explaining and improving the understanding of this phenomenon of eBusiness [10]. At that time, eBusiness start-ups even patented a number of business model innovations, confirming that this was a new locus of innovation that went beyond advanced information and communication technology (ICT) systems and the service itself [11,12]. Since then, the theoretical understanding of business models has advanced in the field of strategic management. Different streams of research have been established with different orientations. For example, McGrath [13] emphasizes a discovery-driven rather than analytical approach in which new insights are created by engaging in significant experimentation and learning. Casadesus-Masanell and Ricart have pointed out that “the exercise of designing new business models is closer to an art than to a science” [14]. Few approaches have appealed more to the abilities of strategic designers than this strategy approach of “modeling, experimentation, prototyping, and discovery” of business models. However, artifact examples of business models are hard to find, and there is a lack of modeling approaches. This study provides a design perspective that pays specific attention to the visual nature and artifact design of a business model for an online service. Our objective is *to analyze and design a business model for precare services*. To design a model, it is vital to have an understanding of care model characteristics. Internet technologies have created new opportunities in health care and changed the way in which actors within these business models interact. The construction of care models is studied in-depth by framing the essential elements of a business model, and design a care model that structures these elements for the particular Internet technology-enabled precare service context.

### Design Challenge

Internet technologies create new opportunities for eHealth service provision and change the way in which actors interact within new models of value exchange. Although the clinical results of eHealth innovations have proved to be very promising, their implementation is not so straightforward. In fact, problems have been encountered in the adoption of most eHealth innovations [15]. To decrease the failure rate of eHealth service innovations, business model innovation should be given high priority at the start of a project and further developed in iterative loops [16]. The main barrier in adopting online service innovations, besides budgetary limitations, is the organizational model; eHealth services appear not to fit with the organizations of the health care providers. At the organizational level, research has found a gap in understanding the network organization needed between health care providers, receivers, and technology-oriented companies [15]. Prior studies, from a design perspective, have explored eHealth services and their business models, and found that most of the value propositions of eHealth services do not match the real needs of medical professionals and clients [8]. When asked to deliberate on how eHealth could enable them to work more effectively, efficiently, and professionally, both general practitioners and specialists first mentioned quality as the most important value of business model innovation in eHealth. In addition, they expect eHealth technologies to enable better, clearer, and easier communication between different health professionals and care organizations, thereby increasing the level of convenience [17]. Whereas current propositions focus on costs and efficiency, health professionals prefer an emphasis on performance quality and convenience from their perspective of professional practice [18]. The design challenge is to define the properties of these values and model the structure and the design of transactions between actors in order to create such values [8].

### Essential Characteristics of Business Model Design

#### Network Structure

For designing a business model, it is essential to understand its characteristics. First, the business model should be an integrative network model, integrating a network organization with network technology [11,12,19]. The source of innovation is the information and communication technology that enables new models of networked business organization. The network organization includes resources from partner organizations, including customers at home and nonprofit organizations [8]. Such an intrafirm network structure of the business model mediates between technology and economic value, which is an important characteristic of constructing a business model [20]. For the design of a business model, the unit of analysis is the network structure that includes social, technical, and economic elements: The social element is the *network organization*, integrated with the technical element of *Internet and mobile technology*, and in exchange with the economic element of *financing*. The network structure is inherent to a business model, and according to Amit and Zott [21], does not relate to one organization, but to strategic networks [22] and connecting across the boundaries of one organization [16,23]. Furthermore, in considering the business model as unit of analysis and design, it is interesting to distinguish it from what a business model is not.  A business model is not a marketing model or only a pricing or revenue model. Nor is its business component in isolation, such as only a value proposition or network structure. Neither is a business model a policy or strategy, such as a corporate strategy, market adoption strategy, or product market strategy. A business model is not even a business process [24]. Although business models are generated for a single firm context, many practitioners used the “business model canvas” [25] for this purpose; the real design challenge is to connect the value propositions and finance in a network structure design [23,26]. The business model canvas frames the standardized elements of a business model. However, just as a SWOT (strengths / weaknesses / opportunities / threats)-canvas does not model a strategy, neither does this canvas model the business model, in the sense of providing an artifact design of the model. The standardized building block elements are neither connected by transactions, nor visualized by a model structure of the network that uniquely identifies the business model. To some extent this canvas appears to be useful, for example, for the analysis and overview of business model elements of the individual firm perspective, but for the design and modeling of a business model, it is not well equipped [8]. The design challenge in our case is using the network structure with an additional toolkit to construct the business models. Second, we extract, as essential elements, from the most cited definition of a business model by Amit and Zott [21], “A business model depicts the content, structure, and governance of transactions designed so as to create value through the exploitation of business opportunities”: (1) “transactions” refer to the network exchanges between organizations; and (2) “value”, which seems to be the most essential element, refers to the purpose of the business model, that is, creating value for customers in transaction with business partners. When we consider the network structure as the unit of analysis of the business model, the core properties that appear to be relevant are the transactions and the value. Teece [27] describes value creation and value delivery as essential properties of a business model. For him, the essence of a business model is in defining the manner by which the enterprise delivers value to customers, entices customers to pay for value, and converts those payments to profit. Chesbrough and Roosenboom [20] indicate that a business model provides a structure of the value chain and describe the position of the firms within the value network. The firms’ organizations and the person representing the organizations are the actors in the value network of service providing. With regard to the modelling challenge of designers, we postulate to model the network structure of actors and value transactions. Using and advancing these essential business model characteristics, the design of a business model can be reframed as, *visually modeling a network structure of actors and value transactions.*

**Figure 1:**
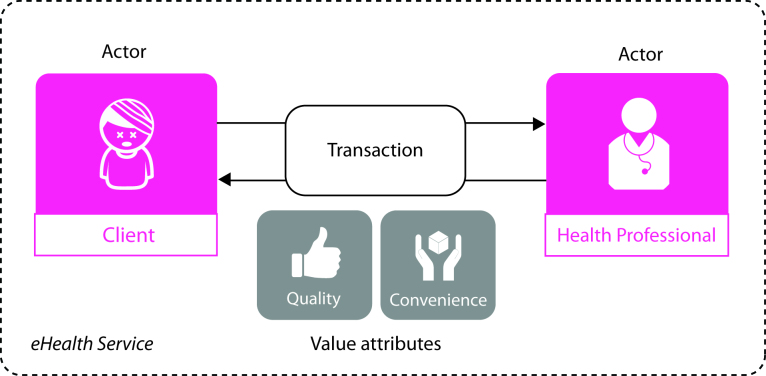
Conceptual framework for analyzing and designing a care model.

The conceptual framework visualizes the essential elements for analyzing and designing a care model (Figure 1 shows this). The framework focuses on the values of convenience and quality for the clients who make use of the eHealth service offered by the health professional. The most essential transaction in an eHealth service proposition that creates value by providing quality and convenience occurs between these two actors, and thus we frame this transaction as the starting point of business model design.

#### Actors

We follow Herzlinger, who suggests putting the client in charge of health care in order to generate more freedom of choice, openness, and transparency in the design of business models [28]. Designing from a client perspective can give insight into the tighter relationship between actors who create and deliver value to the client. The client is therefore placed in the center of the conceptual framework. We define the client actor as, “a person using the services of a professional organization” [29]. These services are provided by the health care professional, the second actor in the conceptual framework. The health professional can be the specialist, the general practitioner, nurses, etc. When designing a business model, additional interactions with other actors need to be investigated further.

#### Transactions

We follow Amit and Zott, who relate transactions to both the economic theory of transactions and the network theory in organizational behavior [21]. The primary, most important transaction in the conceptual framework is the customer value proposition defined by Johnson et al as, “how the health professional creates value for the client by providing a solution for a fundamental problem in a given situation” [30]. By definition, the transactions are reciprocal in nature. In return for the value proposition, the client pays a fee, subscribes to an insurance policy, and pays tax to the government for public health facilities and services. This part of the transaction in the conceptual framework is the monetization, concerning when and how money is raised, as defined by Baden-Fuller and Mangemartin, “Monetization involves more than just pricing (the economists concern), but includes systems determining timings of payments and methods of collecting revenues” [31]. This financial exchange part of the transaction builds on the market innovation approach of Kim and Mauborgne, who analyzed pricing in relation to cost targets and partner capabilities in the value network relations. The types of monetization they introduced include direct selling, leasing, time share, slice-share, and equity payment [32].

#### Value Attributes

Value attributes can be attached to transactions. These attributes—the potential properties of transactions—can add value to both the perceived quality and convenience of the eHealth service for the client. For instance, “quality value” is focused on improving the product or service performance, and “convenience value” is defined as “making products or services more convenient and easier to use” [25]. When designing a business model, it is essential to create value for the customer. Ostenwalder identified three types of customer value. The first, use value, is provided by the actual use of a product or service (eg, driving a car) when its attributes (eg, features, design, support, etc) correspond to the client’s needs and expectations. The second, risk reduction value, reduces the client’s risks (eg, car insurance), such as by alleviating financial fears and providing buy-back guarantees. Insurance contracts provide this value. The third, effort value, makes the client’s life easier (eg, home delivery of groceries); by reducing efforts that are time consuming and/or require specific skills [25]. In eBusiness, this last type of effort value also works the other way around. When the customer fills out the order forms and other administrative details, this reduces the effort of the eBusiness firm. This Internet-enabled efficiency makes it possible to lower the price (cost value) and also provides great potential for lowering the organizational health care cost. In prior research, medical professionals have stated that they favor performance quality and convenience value over cost and efficiency value. The medical professionals showed the least interest in novelty, design, and brand value [8].

**Table 1:** Care model constructs.

Element	Construct	Definition
Actor		In the value network of service providing, the firm, the organization [33], and the person(s) representing the organization.
	Health professional	A professional working at a health care providing service organization, such as the medical specialist, the general practitioner, specialized nurse, etc.
	Client	A person using the services of a professional organization [29].
Transaction		Reciprocal exchange of value. Core transaction, value proposition in return for monetization.
	eHealth value proposition	Providing a solution for a fundamental problem in a given situation [30] of online service providing of health care by Internet- and mobile-based applications and technologies [3].
	Monetization	When and how is money raised? Monetization involves pricing including the systems determining timings of payments and methods of collecting revenues [34]. Such as direct selling, leasing, time share, slice-share, and equity payment [35].
Value attributes		Properties of transactions, that add value to the use, effort experience, and risk reduction [25] of the eHealth service.
	Quality	Value of improving the product or eHealth service performance [25].
	Convenience	Value of making products or services more convenient and easier to use [25].

### Research Question

In this study, we further investigate the construction of business models in depth by framing the essential elements of actors, transactions, and value attributes. In order to design a business model that structures these elements specifically for the Internet technology-enabled precare service context, we address the following research question, “What characteristics of business model design in precare contribute to the convenience and quality of eHealth services provided by a health professional to a client?”.

To deconstruct the service relationship between the client and the health professional, a designerly modeling approach is valuable in locating the different actors, transactions, and value attributes that fit the preferred value proposition focusing on the convenience and quality of eHealth services. These elements of a business model are a steppingstone in the creation of building blocks; compositions of actors, transactions, and attributes that are valuable in the design of business models of eHealth services within the precare phase.

The next section addresses the Methods in which we report on the research method and the toolkit developed for the visual design of business models. Then the Results section provides an empirical analysis of the actors’ perspectives on the business model for a precare service and presents the newly designed business model artifact for this precare service. In the final section, we draw conclusions and discuss the research limitations and suggestions for future research.

## Methods

### Case Study

#### Selection

To arrive at a better in-depth understanding of the business-modeling phenomenon within its real-life context, we adopted a case study method [34]. Case study research, when it is convincingly grounded in the evidence, can generate frame-breaking insights [34]. We selected an innovative eHealth case, PRE (anonymized case name for the start-up organization with an online precare service for living a healthier life), an online precare service that fits the purpose of the research. As a baseline for the point of departure, we define the relationship between the two actors: the client and the health professional (Figure 1). The client actor in a precare context is the person who engages in some type of health-protective behavior [9]. According to Haris and Guten [9], the major concerns are the health beliefs of individuals (clients) and their settings, and the environmental cues to action or characteristics of the health care delivered to them that can be modified in the design of a business model. Health-protective activities include direct contact with a health care professional, the second actor in the conceptual framework. Exploring this transaction relation in practice from different viewpoints, and by further deconstructing the details, helped in the identification of valuable care model characteristics. We used a visualization method to turn the implicit business model into an explicit model. In a structured and visual way, we collected data on the actors, transactions, and performance attributes of the online precare service. We then conducted a within case analysis, including the different perspectives involved in the e-service. Based on the analysis, we constructed the design for the business model.

#### Case PRE

**Figure 2:**
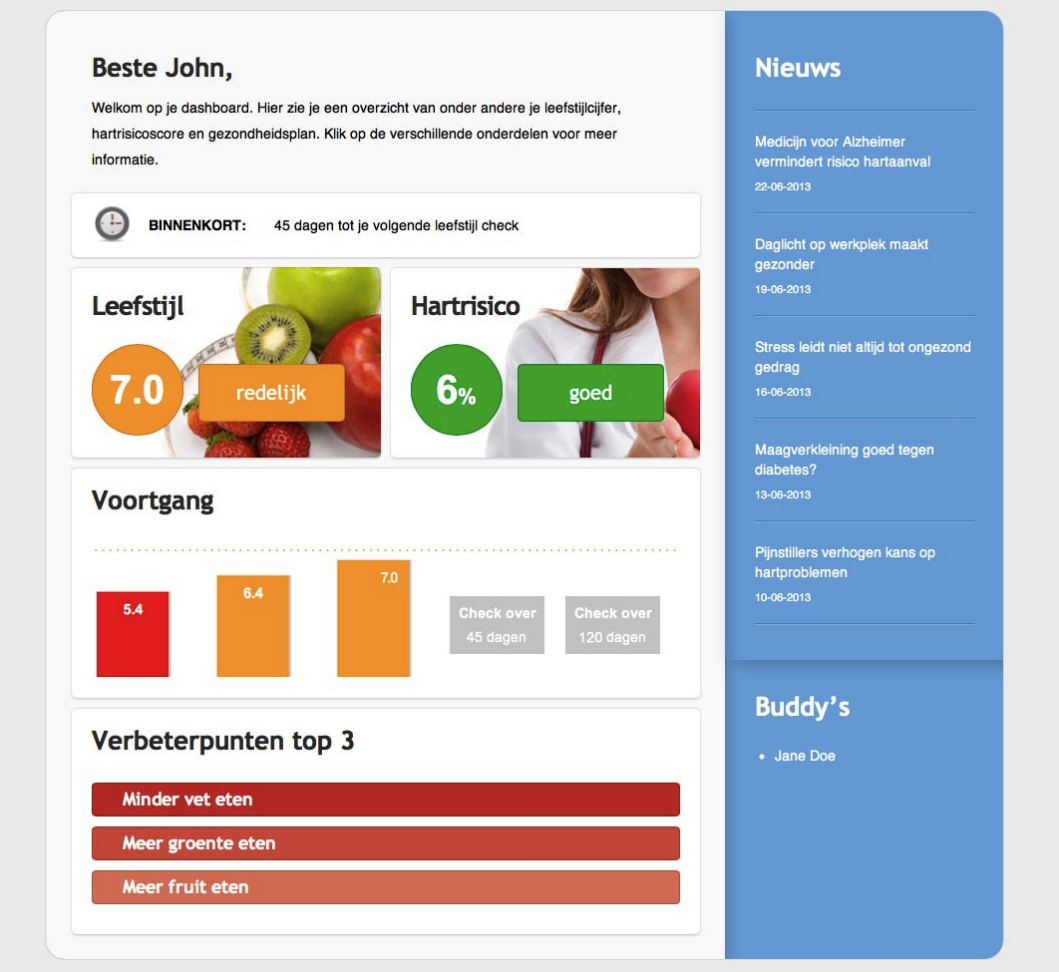
PRE (anonymized case name for the start-up organization with an online precare service for living a healthier life ) Web application service.

PRE is a start-up organization launched by a professional cardiologist. The purpose of PRE is to make clients aware of their lifestyle and heart risk by supporting them with an online service for living a healthier life. On the PRE Web application, a client creates a personal online account and gets feedback information, including a grade for his or her personal lifestyle and a percentage chance of heart failure. These results are based on an online questionnaire (up to 300 questions), and a small physical examination (taking blood for glucose levels). PRE regularly gives advice on how the client can improve his or her lifestyle, and provides the client with a lifestyle score, updated bimonthly (Figure 2 illustrates an example screen of PRE’s Web-based services).

### Data Collection

Purposeful sampling was used to take a closer look at a relevant eHealth situation, the PRE case. An in-depth investigation of different perspectives was conducted in order to study a variety of views, rather than the mean or average. The sample of data collection (Table 2) includes the capturing of the individual perspectives of five respondents involved in the online health protection service. In total, five respondents were consulted, and six visual models were created.

The PRE service has been implemented as a full operational service after a pilot implementation to ensure the quality of the online service. There were two clients that experienced the system for a minimum of two months that were invited to join an interactive session. Furthermore, a cardiologist (owner of PRE), a manager, and a precare specialist participated in an interactive session. The data were collected over a time span of three weeks. With interactive sessions, participants were guided to deconstruct business models associated with the PRE service.

**Table 2:** Sample of participants in interactive sessions.

Network actor	Respondent	Interview	Visual model
PRE care service provider	CEO^a^/cardiologist	1	1
Intermediair	Manager	1	1
Occupational health	Precare specialist	1	2
Client	Client X	1	1
	Client Y	1	1
Total	5	5	6

^a^CEO = Chief Executive Officer

### Visual Business Modeling Method

**Figure 3:**
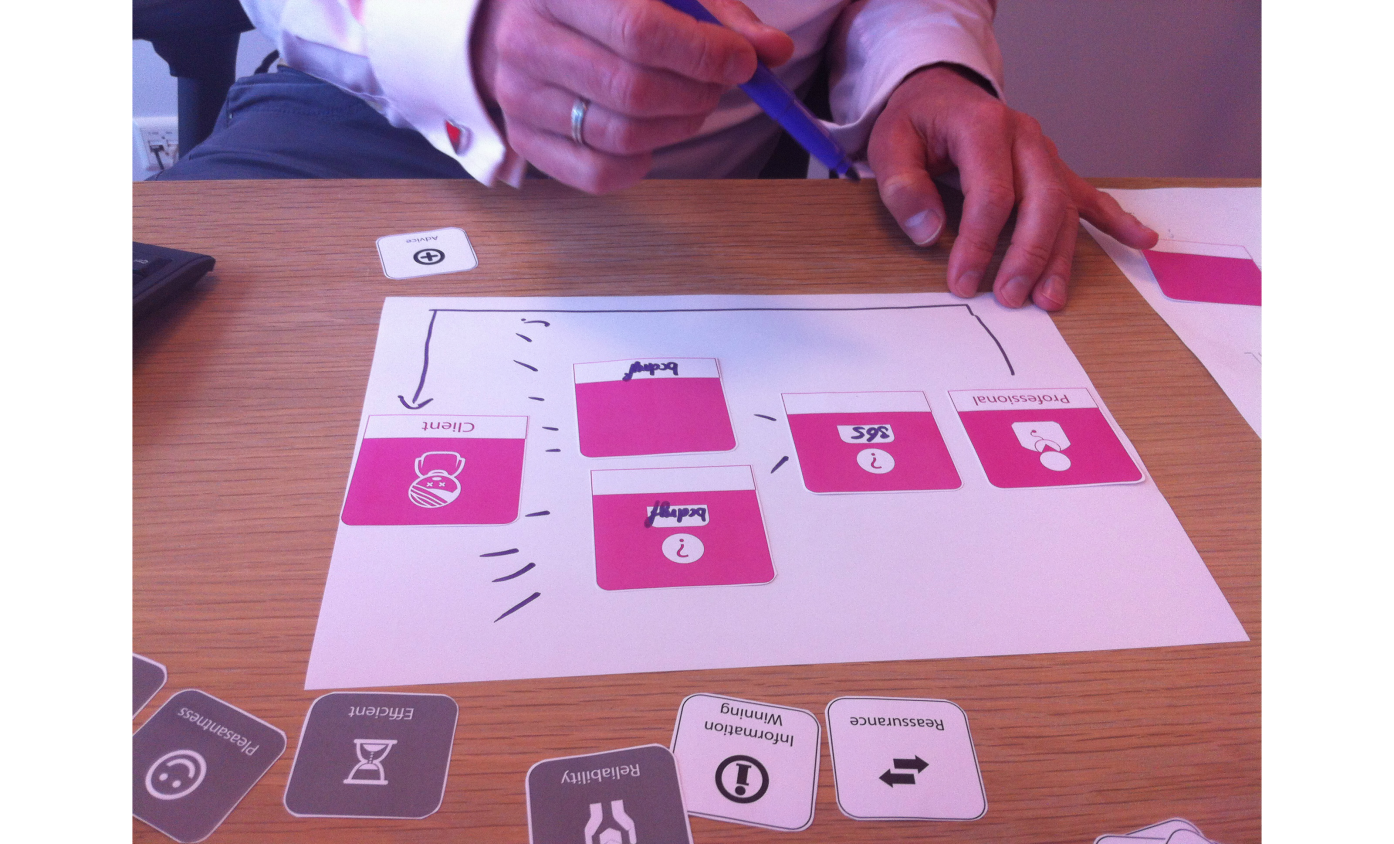
Impression of the use of the visual business modeling method during the interactive sessions.

In the data collection, a visual modeling method was used to support the participant in deconstructing the business model into separate actors, transactions, and valuable attributes. The researchers developed this toolset by combining the toolsets of the visual brainstorm method of the Board of Innovation [36] with the Netmap method used for field studies [33]. This type of actor map toolset is interview-based, and aims to visually capture the connections with many stakeholders and evaluate the types of interactions [8]. The visual business modeling method is a tool that consists of 16 different icons to visualize all types of business ideas, adjusted to the scope of the study by the researchers. The toolset consists of preprinted and “open” cards that can be filled in by the respondent. The preprinted cards are based on the conceptual framework (Figure 1). These cards, starting with two cards for the actors (the client and health professional), contain eight types of transactions and nine attributes that could contribute to the perceived quality and convenience of the eHealth service. Blank cards were included to allow the participants to identify important actors, transactions, or attributes that were not on the tool’s predefined cards. Figure 3 shows one of the participants using the cards during an interactive session.

Each participant was asked to visualize the business models concerning the PRE service by using blank sheets of paper, markers, and the mapping toolset (Figure 3). These interactive sessions were guided by an interview protocol consisting of five subtopics. First, the different actors involved in the business models were allocated to roles by asking the interviewee to pick the corresponding actor cards or write new actors on the blank cards. The second part focused on the connections between those actors, and the third part on the characteristics of these connections, that is, the transactions. Fourth, if applicable, values that contribute to the perceived convenience and quality of the service were ascribed to the related transactions. The visual modeling method made use of red flags to mark the most important attributes. All interviews were recorded. Within-case evidence was acquired by analyzing the records, taking notes, and combining the notes with the created visual models.

### Data Analysis

There were three types of data that were analyzed: (1) visual modeling data, (2) interview data, and (3) documented data. All the types of qualitative data were combined to frame, analyze, and synthesize the business model view of each respondent. The visual business models created by the participants were analyzed by means of a comparative analysis. The in-depth analysis of different perspectives meant that a variety of views, rather than the mean or average, were investigated. By analyzing the different visual models created from the different perspectives, the created models were transcribed into digitalized models. When comparing these models and extracting valuable actors, transactions, and attributes, conclusions were drawn regarding the elements contributing to the convenience and quality of the service. Based on these insights, building blocks were created that can be used in the design of an eHealth service. For the building block design, the actor-transaction toolset was used as a basis for designing the new business model for the precare phase of care.

## Results

### New Identified Actors

The framing of all business model views resulted in the identification of important actors, transactions, and value attributes. In addition to the two client and health professional actors in the conceptual framework, eight new actors were identified, framed with eight different types of transactions and six additional value attributes.

**Figure 4:**
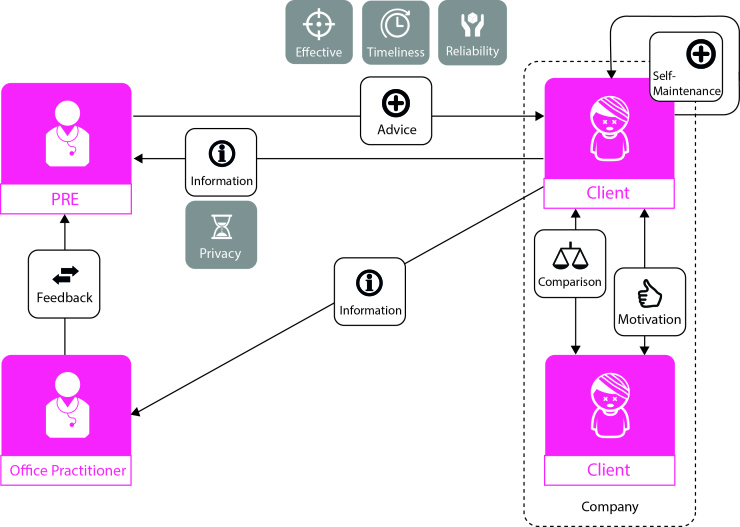
Business model view A from client perspective.

**Figure 5:**
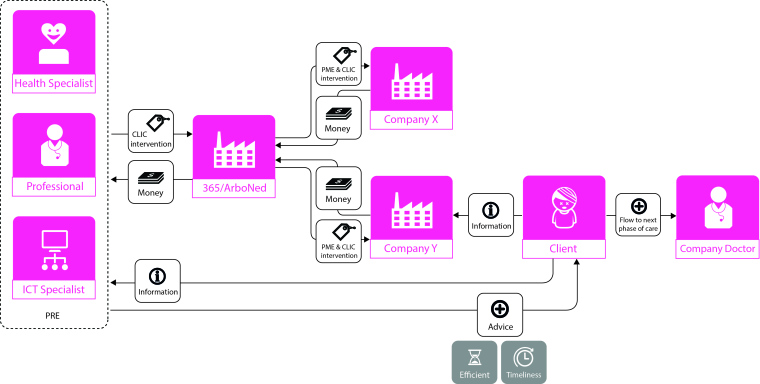
Business model view B from PRE (anonymized case name for the start-up organization with an online precare service for living a healthier life) health professional perspective. PME:preventive medical examinations.

There were three actor perspectives that were synthesized from the six individual business model views. Figures 4 and 5 show the evidence of two of these business model views that have been cocreated and digitalized. The generated insights from the comparative analysis of these visual business models are presented in the following paragraphs. For each of the three perspectives, the new insights are described regarding the type of transactions between newly identified actors, and their perceived valued attributes from online PRE service. The first perspective is from the online service provider, the second from the network coordinator, the intermediate organization in occupational health services, and the third perspective is from the clients working at a company that has contracted the occupational health services.

### Online Service Provider Perspective

#### Overview

The first perspective analyzed in the deconstruction of the PRE care model is the perspective of the online service provider. The health care professional is the developer and owner of the service. Table 3 categorizes the actors, transactions, and value attributes of the care model that are of importance for the online service provider.

**Table 3:** Online service provider overview of actors, transactions, and value attributes.

Actors	Transactions	Value attributes
ICT specialist	Online precare service, PRE health protection	
Health specialist	Recommendations	Timeliness
Intermediate organization, 365/ArboNed^a^	License for precare software products	Efficient

#### Actors

From the health professional perspective, three new actors were identified as an essential part in the care model. These essential actors are key developers and a partner to deliver the service to the client. First, the *ICT specialist* was mentioned as an important actor in the development of the service. He transforms the regular precare service into a new online service. Second, a *health specialist* contributes to the professionalization of the content of the health recommendations. Third, as most important customer actor was identified, the 365/ArboNed organization, this is the *intermediate organization* dedicated to occupational health and safety of employees, the 365/ArboNed organization. To extend the reach of the service, PRE has partnered with this organization. For 365/ArboNed, PRE is a unique selling point, increasing the quality of the services offered to the companies within its network. 365/ArboNed, situated in the Netherlands, is “an occupational health service that is contracted by more than 72,000 companies nationwide to provide their employees with occupational health care” [37].

#### Transactions

##### 1. Online precare service, health protection recommendations

In framing the relation between the client and the health professional, PRE is an online precare service providing professional lifestyle advice. The PRE service consists of a “flow” of digital triggers and recommendations, including weekly tips and tricks, video messages from the health professional, and extensive bimonthly advice. This transaction in which the service provides health protection recommendations only takes place if the client’s heart risk and lifestyle score are below seven on a scale of ten. These scores are based on the client’s responses to several questions regarding his or her lifestyle, which are included in the yearly Preventive Medical Examinations (PME) of the occupational health service. Employers in the Netherlands are legally obligated to offer PME to their employees.

##### 2. License for precare software products

The software products in the transaction between the health professional and the intermediate organization provide the basis for the pricing and licensing of the business model. The precare software modules are paid for by the intermediate organization with a license fee. The license covers the use of the online heart risk and lifestyle assessment services (including the estimation of scores and recommendation messages) from PRE software modules in its online PME questionnaire. The precare intervention service is an additional option in the contract provided by the intermediate organization to the companies.

#### Value Attributes

The aim of the health professional in the design of the PRE service is to disseminate lifestyle advice to as many clients as possible. Therefore, *efficiency* is one of the main attributes of the online precare service transaction. In addition, the health professional noted the importance of the *timeliness* of the service, which provides a continuous flow of advice to the client by means of weekly tips and tricks, video messages, and recurrent questionnaires. The health professional considers the digital characteristics of an online eHealth service as an opportunity to create personal, automated advice in precare. The system behind the service constructs this advice by combining short text passages—entered in the system by the professional—based on the outcome of the PRE questionnaire.

### Network Coordinator Perspective

#### Overview

The second perspective, from which insights are derived, is the perspective of the coordinator of the PRE service within the 365/ArboNed network of business-to-business customers. In protecting health within the firm environment, the network coordinator pointed out the existence of two different situations: (1) the normal situation when the client is at low risk and thus no intervention is needed, and (2) the service situation where intervention from PRE is needed (Table 4).

#### Actors

From the network coordinator perspective**,** zooming in on how the organization delivers the online precare service to the clients identified three additional new actors. First, *the marketing department of 365/ArboNed* is an actor that integrates the precare service in service package contracts for the second identified actor, *the companies within its network.* Third*,* the occupational health service employs a precare specialist, a so-called *vitality and health expert*. This actor has a direct relation with PRE regarding the information and content of the precare service, and has a coaching relation with certain clients, with whom this dedicated precare specialist holds face-to-face meetings. This last actor evidences that the delivery of the online service on a computer device comes with personal contact for a certain group of clients requiring coaching on precare. This situation corresponds to the situation when, due to high heart risk and a low lifestyle score, PRE intervention is required. In this situation, another new actor is involved, *the office practitioner,* who is involved when a PRE intervention is started. The client’s heart risk and lifestyle scores are complemented with heart rate and cholesterol level measurements. The client, therefore, needs to schedule an appointment with his or her office practitioner to take a blood sample.

**Table 4:** Network coordinator’s overview of actors, transactions, and value attributes.

Actors	Transactions	Value attributes
Marketing department	Service contract	Reliability
Companies within network	Software products transaction, PME digital feedback report	Privacy
Office practitioner	Check-up measurement	Availability
Vitality health specialist	Personal coaching	Privacy
	Product support feedback	Personal interaction

#### Transactions

##### 3. Service contract for precare services and software products transaction

The relation between the health professional and the intermediate organization involves a value transaction, as the software modules are paid with a license fee. The precare software products are embedded in the PME. 365/ArboNed has incorporated the heart risk and lifestyle scores in the PME assessment as a unique selling point in its service package proposition to companies. In addition to providing feedback to the client regarding the outcome of the test, 365/ArboNed gives feedback concerning the preventive medical results to his or her employer. The employer can optionally request a report on the lifestyle scores of different departments in the company.

Within the company contract packages, the PRE intervention service based on employees’ heart risk and lifestyle scores is optional. When a company chooses to include the PRE intervention service in its contract with 365/ArboNed, and one of the clients in its employ needs intervention coaching—when the PME results show high risk—direct contact is made between the client and PRE. This transaction is described in the health professional’s perspective.

##### 4. Preventive Medical Examinations Digital Feedback Report

The PME is a questionnaire that consists of three hundred questions assessing a client’s everyday lifestyle and behavior. The client receives a summary of the outcome of the questionnaire in the form of a digital feedback report. If the client’s lifestyle score is below seven, and his or her employer has included the PRE service in its precare service package, a PRE intervention is initiated. Besides the intervention transaction between PRE and the client, this also entails a personal start-up meeting between the precare specialist and the client, and an appointment with the office practitioner.

##### 5. Personal coaching and check-up measurement

If the client’s lifestyle score is below seven on a scale of ten, a PRE intervention transaction will be started. To establish this intervention transaction, two new transactions are initiated. First, direct and personal contact between the precare specialist and the client is established, and second, between the precare specialist and the office practitioner. In a face-to-face start-up meeting, the vitality and health expert (VHE) can support the advice the client receives from PRE, and motivate the client to improve his or her lifestyle. The office practitioner has to measure the client’s heart rate and take a blood sample to determine cholesterol levels.

##### 6. Product support feedback transaction

The VHE provides the developers of the precare service (the PRE team) with feedback in order to improve the overall quality of the service. The feedback is related to the process coordination of the PRE service, and how the ICT architecture behind the service can be improved. Although this transaction can be valuable in the design of any eHealth service, in this case the experience of the VHE with PRE is of importance because the incorporation of PRE in the PME’s of 365/ArboNed only started toward the end of the year in 2012.

#### Value Attributes

In addition to the attributes of efficiency and timeliness, the managers of the intermediate organization came up with four new value attributes in the care model of precare services. First, the client’s *privacy* needs to be ensured when entering personal information into an online system. Second, within the digital feedback report transaction, *reliability* is a necessary attribute, contributing to how the client perceives the quality and convenience of the service. Third, in the transaction in which the health professional intervene, the *availability* of the advice has been identified as a valuable element that contributes to the perceived usability of the precare service. The *personal interaction* of the face-to-face start-up meetings increases the convenience of the service. In addition to these meetings with the vitality health specialist, this type of direct contact also takes place in the check-up measurement appointment with the office practitioner.

### Clients’ Perspective

#### Overview

To explore the transactions from the clients’ point of view, two clients, client X and client Y, were interviewed. The created care models showed many similarities, and only differed on a few attributes of the transactions between PRE and the client (Table 5).

#### Actors

From the client perspective, one new group of actors was identified. This group of actors comprises the other clients making use of the professional intervention service. These “coclients” can vary from familiar colleagues to anonymous users. Close contact between coclients can help motivate clients to improve their lifestyle and their heart risk and lifestyle scores. According to client Y, the business-related context of the interaction among clients provides a supportive boost in sharing and comparing heart risk percentages and lifestyle scores, information that is generally perceived as highly personal.

**Table 5:** Client’s overview of actors, transactions, and value attributes.

Actors	Transactions	Value attributes
Coclients	Self-management	Pleasantness, reliability, privacy
	Flow to next care stage	Timeliness, effectiveness

#### Transactions

##### 7. Self-management of health protection by the client

In the deconstruction of the care model by the clients, self-management was identified as a new transaction. Self-management refers to the interventions, training, and skills by which patients can effectively take care of themselves, and learn how to do so. In contrast to the other transactions, which involve an exchange of activities between two actors, this transaction is the client’s relation with himself or herself in the self-management of his or her state of health. The earlier identified transactions of a personal tailored feedback report, a professional intervention service, personal coaching and check-up measurement, and online social sharing serve to support the self-management of the client.

##### 8. Online social sharing transaction

In the interaction with other clients, comparison and motivation were mentioned when the interviewed clients deliberated about their health-protection activities. Clients compare their scores and thereby motivate each other.

#### Value Attributes

Within the transactions between the PRE online service and the client, client X identified *pleasantness*, *timeliness*, and *reliability* as the core value attributes contributing to self-management and the quality and convenience of the intervention service. Like client X, client Y stated that timeliness and reliability are core value attributes in this transaction. *Timeliness* is a result of the continuous flow of information that keeps encouraging the client to adjust his or her lifestyle. However, instead of pleasantness, client Y stressed *effectiveness* as another element contributing to the perceived convenience and quality of PRE. In addition, client Y emphasized that guaranteeing *privacy* is critical when entering personal information into the online system. For this client, the privacy issue in the PRE service is solved by the involvement of a professional health care provider in the development of the service. Both clients perceived PRE as a reliable service for the same reason, because a professional cardiologist developed it.

### Design

#### Synthesis

When comparing the different business models created from the different actor perspectives, conclusions can be drawn regarding the elements of a business model that contribute to the convenience and quality of the eHealth service in precare. These elements can be divided into actors, transactions, and attributes. Only when both actors confirmed the transactions or relating attributes took place between them, the transactions or relating attributes were validated.

#### Actors

Concerning the positioning of actors, three major insights emerged from the analysis results. First, a professional such as a health care provider should be involved in the development of an eHealth service in order to ensure that the client feels secure when entering personal information into an online system. Moreover, the professional qualifications of the involved health care provider ensure the reliability of the advice provided to the client. Second, an intermediate organization with a large network can be valuable in connecting the provided service with potential clients. Third, face-to-face contact, such as with the precare specialist or office practitioner, has an influence on how the client perceives the quality and convenience of the service. This direct contact serves to support the client’s self-management. The actual presence of the precare specialist is important, since the transaction between this actor and the client, the actual support, has a lesser influence in the minds of the client’s on the perception of the service.

#### Transactions

From the deconstructed care models, we derived three insights with regard to the transactions contributing to a valuable eHealth service. The first transaction is the aim of any eHealth service in precare, and entails self-management of the client. This transaction is supported by the second interaction between the client and the eHealth service. This connection needs to consist of a flow of information from the client to the service, and, based on this information, a flow of advice from the service to the client. A distinctive characteristic of the interaction between the client and an eHealth service is the digital nature of both flows. Another transaction positively influencing the self-management of the client is the motivational boost provided by interaction with other clients when they compare scores and share tips.

#### Value Attributes

Finally, insights can be derived from the attributes the participants attached to the transactions. Based on the results, we can conclude that four attributes contribute to the convenience and quality of online services, and thereby influence the design of eHealth services. These attributes are the client’s privacy when providing the service with personal information, the reliability of the advice received by the client, the timeliness of the advice received by the client, and the preferred type of contact with a precare specialist. Both privacy and reliability are perceived values of the client when a professional health care provider is involved in the development of an eHealth service. Direct contact with a precare specialist or office practitioner positively influences the perceived quality and convenience of an eHealth service. Timeliness involves the time management of the advice transaction between service and client; a continuous flow contributes to the convenience and quality of the service.

### Constructing Building Blocks

#### Overview

**Figure 6:**
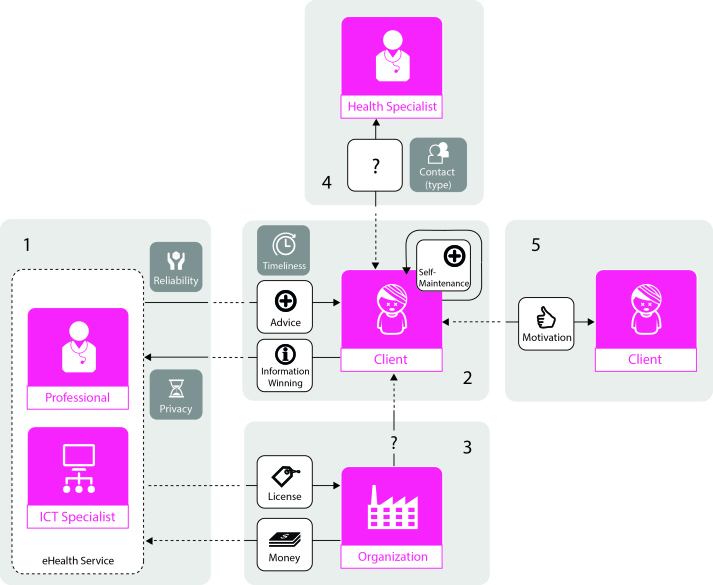
The five building blocks integrated in the business model design for eHealth services in precare. ICT: information and communication technology.

Based on these insights, we constructed five building blocks. These building blocks—elements of a business model consisting of actors, transactions, and value attributes—contribute to the convenience and quality of an eHealth service. Together these building blocks form a business model that can be used in the design of any eHealth service in the precare phase. Figure 6 shows this model, consisting of the five building blocks.

#### Building Block 1

The involvement of a health professional, involving a professional health care provider in the development of an eHealth service will ensure privacy and reliability in the transactions between client and service.

#### Building Block 2

Interaction with the health professional, information flow from the client to the service, and a continuous flow of advice back to the client are needed in accomplishing self-management of the client.

#### Building Block 3

Coordinating network organization, involving an intermediate organization with a large network can assist in extending the service’s reach, connecting the service with potential clients. However, on the basis of the results, no conclusions can be drawn concerning the nature of the transaction between the organization and the client.

#### Building Block 4

Direct contact, face-to-face contact between the client and a precare specialist supports self-management of the client and his or her perception of the eHealth service. The type of transaction has less influence, and is therefore labeled with a question mark.

#### Building Block 5

Interaction with other clients, the presence of other clients making use of the same eHealth service has the potential to motivate the client and support his or her self-management.

In order to design an effective business model for eHealth service solutions, all building blocks need to be integrated. All building blocks are of equal importance; the combinations of actors, transactions, and attributes all contribute to the convenience and quality of an eHealth service.

## Discussion

### Principal Findings

Taking up the design challenge of creating an innovative business model for an eHealth service, we framed the characteristics of a business model and constructed a care model that structures the actors, transactions, and value attributes for the particular context of an online precare service. Based on in-depth insights on which value exchanges between a health professional and a client are important, we constructed the building blocks of a care model for precare eHealth services. From five actor perspectives, we identified and defined the care model actors, transactions, and attributes that contribute to the perceived convenience and quality of this particular eHealth service. The generated insights and design contribute to this situation, but also have broader relevance for comparable online eHealth services. As more generic implications for the design and implementation of eHealth innovations, we propose to: (1) involve a health professional, since this will ensure privacy and reliability in the transactions between the client and the service; (2) provide an online flow of information with regular interactions to the client in order to stimulate self-management of personal health; (3) involve an intermediate organization with a large customer base to extend the service’s reach; (4) involve a service-dedicated health expert for personal face-to-face contact with clients in order to ensure and increase the perceived quality of the eHealth service; and (5) include social interaction with other clients of the online service with a view to motivating and supporting the self-management of personal health.

As more generic implications for the design and implementation of *care models*, we propose to: (1) investigate role perspectives, first by uncovering all the actors involved, then analyze, together with each actor, their own positioning in exchange with other actors, unraveling the value exchange of the transactions between actors in the network; (2) visualize the care model situation guided by the visual modeling toolkit, which helped actors to structure their thoughts, and provide knowledge on organizational conditions that are relevant for the design of the care model; (3) design building blocks from a deconstruction of the (visual) role perspectives on an organizational network model of value exchange; and (4) create artifacts of care model designs to communicate about the implementation of business model innovation for eHealth services (involve strategic designers).

### Limitations and Further Research Suggestions

Although the study was executed in depth and from multiple role perspectives, the choice of a single case study method comes with limitations. These include the two clients who used the precare service for different lengths of time; one client tested the service for two months, and the other for one year. Also both clients had a low heart failure risk and high lifestyle score. These differences influenced their perception of the service, but overall the ways in which they visualized the care models had more commonalities then differences. A first suggestion for further research is to conduct a multiple case study with more interviewees per role perspective. A second research suggestion is related to the visual business modeling method, in which the participants could freely modify and add actor, transaction, and attribute cards. Permitting this degree of flexibility and creativity in codesigning could have positively affected the participants’ openness to the implementation of the care model innovation. This research by design method required reflection on action, demonstrated with the deconstruction part of this study. Further research experiments on the visual elements in the business modelling kit and on the codesigning situation can bring the modelling method to a next level of intervention. Another relevant direction for further research would be to quantitatively validate the influence of the separate building blocks on the convenience and quality of the service, in contribution to the knowledge field of care models in eHealth. Also further quantitative research on monetization (eg, cost structures and revenue streams) is worthwhile to investigate. Furthermore, an interesting related avenue for further research in complex value network structures is the in-depth analysis to overcome the key barriers for the integration and adoption of eHealth services. Concerning the monetization barriers, such as willingness to pay, issue of having no financial reimbursement structure for eHealth services, requires further research. A final consideration in this case study relates to the venture context. The eHealth service was studied in a business start-up context. The importance of building blocks might change or new blocks could appear in different phases of the service’s life cycle. Understanding the developments a business model in eHealth goes through enables forward-looking design of business models for eHealth services. In addition, our final research suggestion is to study the influence of the different stages of a service’s life cycle on the design of its business model.

### Conclusions

This study provides an overall business model that is informative and serves as a source of inspiration for the creation of eHealth services in the precare phase of care that provide convenience and quality to the end user. By using a visual modeling method in codesign with the actors involved, the essential actors, transactions, and value attributes of a business model were discovered in the context of the PRE case study. We revealed eight actors in the business model of the precare service. Essential for providing the service are: the intermediary network coordinator connecting companies, the service dedicated ICT-specialists, and the service dedicated health specialist. In the transactions we found a certain type of contract, such as a license contract and service contracts for precare services and software products. In addition to the efficiency, quality, and convenience value attributes, important value attributes appeared to be: (1) timeliness, (2) privacy and credibility, (3) availability, (4) pleasantness, and (5) social interaction. As such, the final business model emphasizes the importance of real-time contact between the client and a health care provider in online interactive intervention programs. Moreover, larger groups of clients could be treated in the precare stage at the same time, thereby educating and helping clients to self-manage healthier behavior, while also stimulating dialogue and support between clients.

## Abbreviations

ICT: information and communication technology
PME: preventive medical examinations
PRE: anonymized case name for the start-up organization with an online precare service for living a healthier life
VHE: vitality and health expert
